# Diversity and distribution of fish fauna of upstream and downstream areas at Koto Panjang Reservoir, Riau Province, Indonesia

**DOI:** 10.12688/f1000research.19679.2

**Published:** 2020-02-05

**Authors:** Netti Aryani, Indra Suharman, Azrita, Hafrijal Syandri, Ainul Mardiah

**Affiliations:** 1 Department of Aquaculture, Faculty of Fisheries and Marine Science, Universitas Riau, Pekanbaru, Riau, 28293, Indonesia; 2 Department of Aquaculture, Faculty of Fisheries and Marine Science, Universitas Bung Hatta, Padang, 25133, Indonesia; 3 Department of Aquaculture, Faculty of Marine and Fisheries Science, Nahdlatul Ulama University of West Sumatra, Padang, 25176, Indonesia

**Keywords:** Ichthyodiversity, shannon’s index, evenness, exotic, reservoir, river

## Abstract

**Background**: The capture fishery sectors in the river and reservoir play an important role in the Indonesian economy through increased income and diversification of livelihoods. The present study was conducted to ascertain fish diversity and their distribution pattern in the upstream and downstream areas of Koto Panjang Reservoir, Riau Province-Indonesia.

**Methods**: Fish samples were collected for a period of 12 months using a variety of fish nets at four sites; Koto Mesjid (KM) and Batu Bersurat (BB), located in the upstream area of Koto Panjang Reservoir and Rantau Berangin (RB) and Kuok (KK), located in the downstream area of Koto Panjang Reservoir. Data obtained were analyzed using standard taxonomic keys based on morphometric characters.

**Results**: A total of 44 species belonging 19 families and 33 genera were recorded in the study area. Alpha diversity indices showed that fish diversity in this area was quite high (Shannon’s index = 2.10 and Simpson-D = 0.21) and evenness was low (evenness H/S =0.19). The ﬁsh in KM and BB sites (upstream) were from eight and 11 families, respectively. In RB and KK sites (downstream), fish were from 16 and 15 families, respectively. In KM, BB, RB and KK sites, the dominant family was Cyprinidae, comprising 33.45%, 50.95%, 43.04% and 39.35% of all fish caught at each site, respectively. Exotic species, especially Nile tilapia, were 20.15%, 14.11%, 5.62%, and 5.34%, respectively. Some differences were also noted between the upstream and downstream reservoirs, with a slight increase in exotic species in the upstream reservoir over the study period (from 11.39% vs. 34.66%), corresponding to decrease of native species (from 88.61% vs. 65.34%).

**Conclusion** **s**: The diversity and distribution of fish fauna were varied in upstream and downstream areas of Koto Panjang Reservoir. The exotic species were found to be dominant in the upstream reservoir areas.

## Introduction

The capture fishery and aquaculture sectors play an important role in the Indonesian economy through increased income, diversification of livelihoods, supply of animal protein, and foreign exchange earnings. In 2018, the total fishery production of Indonesia was 23.186,442 metric tons, of which 467,821 metric tons was obtained from inland capture fisheries, 6,603,631 metric tons was obtained from marine fisheries, and 16,114,991 metric tons was obtained from aquaculture fisheries production ^1^. A total of 1,300 fish, including 40 endemic species are known to inhabit in the freshwaters of Indonesia, with 16 exotic species recorded in Indonesia ^2^. The production from the inland capture fisheries of Indonesia comes from wetlands (rivers, lakes, swamps, oxbow lakes, floodplains, etc). In Riau Province, one of the rivers that produce freshwater fish from capture fishery is the Kampar Kanan river. Fithra and Siregar ^3^ found 54 species in Kampar Kanan River, while Aryani ^4^ reported 40 species. At present, major Cyprinidae species such as *Leptobarbus hoevenii*, *Osteochilus haselti* and *Rasbora argyrotaenia,* along with exotic species, such as common carp ( *Cyprinus carpio*) and Nile tilapia ( *Oreochromis niloticus*), are the available species of fish at local food markets.

According to Mulyadi ^5^, Kampar Kanan river is one of the largest rivers in Riau Province. It is approximately 213.5 km long and between 125 to 143 m wide, with significant capture fishery potential. Since 1996, this river has been dammed into a reservoir (Koto Panjang Reservoir) for the operation of a 114 MW hydroelectric power plant. The dam height is 96 m and located at altitude of 85 m above sea level ^5^, and at the geographical position 0°17'23.76˝N and 100°52'53.39˝E. However, at the location of the dam, there is no fishway. The abiotic and biotic characteristics of river ecosystems can be affected by the construction of dams. These conditions have an impact on mortality and failure of fish migration ^6– 9^. The hydrologic regime of streams changing from lotic to lentic can influence the water retention in the reservoir. In general, the lentic condition causes a decrease in native species and then an increase in exotic species ^10, 11^. Furthermore, the degradation of aquatic fauna habitats can be caused by an increase in homogeneity of water channels, which has an effect on the seasonal flow variability of river ^12^. The reduction in river runoff also affects the habitat and distribution of fish fauna.

On the other hand, there are serious threats to the original fish biodiversity in the downstream and upstream regions of the reservoir due to the dam of the hydroelectric power plant, such as sand mining in river, land use change and aquaculture activity with cages, which can affect the depth of river water, food availability, and breeding sites. Amadi *et al*. ^13^ state that original biodiversity can be eroded by habitat degradation and alien species impact. Meanwhile, aquaculture heavily impacts the structure and diversity of local fish communities ^14, 15^. Hence, it was essential to study fish diversity continuously in different ecosystem areas, including upstream and downstream areas at Koto Panjang Reservoir, Riau Province, Indonesia.

## Methods

### Ethical statement

There are no required permits from the government of the Republic of Indonesia to capture the species in this study in the upstream and downstream regions of Koto Panjang Reservoir. The study was funded by LPPM (Research and Community Service) University of Riau under Directorate of Research and Community Service, Ministry of Research Technology and Higher Education Republic of Indonesia with contract no. 767/UN.19.5.1.3/PT.01.03/2018. This grant included ethical approval and permits to collect fish samples including native species (endangered and non-endangered) and exotic fish species. Specimens of fish species categorized as non-endangered and exotic were killed once caught. Endangered fish species ( *Hemibagrus wyckii)* were returned to the river in good condition following analysis in the field. All efforts were made to ameliorate any animal suffering through anaesthetizing fish with ice water before euthanization.

### Sampling sites and methods of specimen collection

Fish sampling was carried out from January to December 2018 at four sites, namely, Koto Mesjid (KM) and Batu Bersurat (BB) (upstream reservoir), Rantau Berangin (RB) and Kuok (KK) (downstream reservoir) ( Table 1). Fish samples were randomly collected from the study area using traditional fishing gear (e.g. traps nets, cast nets, gill nets, drag nets, and fishing poles). Data was collected once a month at each site and five pieces of fishing gear were in operation at any one time.

**Table 1.** Sampling sites in the upstream and downstream areas at Koto Panjang Reservoir.

Sampling Site	Site Code	Areas	Distance from on Dam position (km)	Latitude	Longitude
Koto Mesjid	KM	Upstream Reservoir	1.2	0°17'06.92˝N	100°52'31.31˝E
Batu Bersurat	BB	Upstream Reservoir	15.5	0°20'12.30˝N	100°44'27.26˝E
Rantau Berangin	RB	Downstream Reservoir	1.2	0°17'59.79˝N	101°54'47.19˝E
Kuok	TB	Downstream Reservoir	15.5	0°23'26.88˝N	101°25'50.64˝E

Trap nets (local name *bubu*) are made from bamboo woven with rattan and have a cylindrical front with a diameter of 80 cm and cone-shaped back, with a length of two meters. Chicken intestine was placed inside the gear as bait. This gear was used between the hours of 18:00 and 06:00 at the bottom of river and reservoir to catch demersal fish such as Bagridae, Pangasidae, Gobitidae, Claridae, Anabantidae, Belontiidae.

Cast nets (local name *Jala*) are a type of active fishing gear made from string, with a length of 2.5 meters and mesh size of 1.5 inches. This gear was used on the river and reservoir sides by fishermen using canoes. This gear was operated during the day from 06.00 until 10.00. The purpose of cast nets is to catch the family of Cyprinidae, Osphronemidae, Notopteridae and Cichlidae.

Gill nets (local name *jaring insang*) are made of rectangular monofilament yarn, are 60 meters in length and 8 meters in depth, with a 2.5 inch mesh size. These were operated passively and transversely on the surface of river from 18.00 until 06.00 to catch pelagic fish such as Cyprinidae, Osphronemidae, Notopteridae and Cichlidae.

A drag net (local name *belad*) is a passive fishing device made from nylon net material with a diameter of 0.15 mm and a mesh size of 0.5 inches. This gear is assisted by bamboo or wood as a cantilever, with a height of 2.5 meters and a length of 100 meters, which was placed parallel to the river coastline from 18.00 until 06.00. The purpose of the drag net is to catch the family of Bagridae, Pangasidae, Gobitidae, Claridae, Anabantidae, Belontiidae and Siluridae.

The fishing pole (local name *rawai*) used consisted of a main line with a length of 50 to 100 cm and a distance from one branch line to another of 1.5 meters. One fishing pole has hooks ranging from 20 to 30 pieces and the hooks are size no. 15. The fishing pole was operated passively on the river bottom between the hours of 18.00 and 06.00 and used chicken intestine as bait. The main purpose of fishing pole is to catch the family of Bagridae, Tetradontidae, Pangasidae and Channidae.

Samples were classified as endangered, non-endangered and exotic fish species based on the categories described by Kottelat and Whitten ^16^. Once caught, fish were anaesthetized in ice water at a temperature of 5°C. Euthanization was achieved by piercing part of the brain of the fish. Samples were given an intra-peritoneal injection prior to store in a formalin solution. Smaller specimens were stored directly in 5% formalin solution, while the larger specimens were stored in 10% formalin solution. Specimens that were categorized as non-endangered were transported in a cold box (10 °C) to the Fish Biology and Ichthyology Laboratory, Department of the Aquaculture, Riau University for measurement of specimen length, weight, and morphometric characteristics. Endangered fish species such as *Hemibagrus wyckii* were analyzed and measured in the field. Then, the same fish was returned to the river in good condition. The length, body weight and morphometric characteristics were only collected for 10 individual specimens from each species.

Samples from each site were separately packed in labeled plastics jars according to date, site, time, and locality. Each specimen was labeled with a specific number manually. Classification and taxonomic identification of the sampled specimens was completed using standard keys ^17, 18^ on the basis of morphometric and meristic characters.

### Data analysis

The data of different species for abundance and occurrence was calculated for species richness (S), Shannon diversity Index (H’), Simpson diversity index (D), evenness (H/S) and Sorenson’s coefficient (CC) ^19– 21^ using Microsoft Excel 2010 (version 14.0). The accuracy of the data and results were verified by applying all the diversity indices separately according to sampling months and sampling sites.

## Results

### Monthly occurrence of fish fauna in the upstream and downstream areas at Koto Panjang Reservoir

During the study, forty-four different species of fish were collected from the study area. A total of 8017 specimens of fish were collected from four sites ^20^. The details of the fish species collected on a monthly basis for the period of one year (January to December 2018) are presented in Table 2. The highest number of fish collected during one month was collected during August 2018 (873 specimens), followed by the months of September > July > June > October > May > November > April > March > December > February > January.

**Table 2.** Ichthyodiversity in the upstream and downstream regions of Koto Panjang Reservoir in January to December 2018.

Family/species	Jan	Feb	Mar	Apr	May	Jun	Jul	Aug	Sep	Oct	Nov	Dec	Total
**Cyprinidae**													
*Barbodes schwanifeldi*	20	25	30	39	40	42	45	50	57	49	40	35	472
*Crossocheilus oblongus*	3	5	3	6	5	7	8	12	15	8	4	5	81
*Crossocheilus langei*	0	0	4	4	8	5	6	8	7	5	3	0	50
*Labiobarbus festifus*	8	12	10	9	13	15	15	17	21	25	12	11	168
*Cyclocheilichthys apogon*	3	3	2	5	10	15	18	14	11	8	5	4	98
*Hampala macrolepidota*	0	0	1	1	0	2	3	2	4	1	1	1	16
*Osteochilus hasselti*	14	16	20	36	30	39	42	40	40	34	29	25	365
*Osteochilus schlegeli*	0	0	1	2	5	6	5	8	2	3	2	2	36
*Osteochilus vittatus*	23	25	20	18	28	21	15	23	26	18	13	10	240
*Osteochilus pleurotaenia*	15	20	23	29	34	30	35	29	27	22	15	12	291
*Oxygaster anomalura*	6	7	12	13	9	15	15	17	13	11	9	7	134
*Puntioplites bulu*	30	31	35	29	28	35	30	24	25	19	32	28	346
*Rasbora argyrotaenia*	60	40	50	55	60	65	75	70	65	60	52	50	702
*Thynnichthys polilepis*	15	18	21	25	19	24	26	22	19	21	20	15	245
*Leptobarbus hoevenii*	0	1	2	3	2	5	4	3	3	2	1	1	27
*Cyprinus carpio*	0	0	1	3	3	5	2	2	3	1	1	0	21
**Bagridae**													
*Hemibagrus nemurus*	32	34	35	38	40	43	38	40	51	58	53	35	497
*Hemibagrus wyckii*	0	0	0	0	1	1	2	2	1	0	0	0	7
*Mystus nigriceps*	23	25	22	26	28	30	31	38	32	25	28	20	328
*Mystus micracanthus*	0	0	0	0	1	5	4	4	3	1	0	1	19
*Claridae*													
*Clarias teijsmanni*	12	15	14	18	21	22	26	29	22	23	17	16	235
**Pangasidae**													
*Pangasius pangasius*	0	0	0	0	1	1	1	1	1	1	1	1	8
*Pangasianodan hypophthalmus*	0	1	2	4	5	5	8	3	2	3	2	2	37
**Siluridae**													
*Ompok hypophthalmus*	45	42	45	48	43	51	55	50	45	41	38	35	538
*Wallago leerii*	10	12	18	23	10	14	24	20	19	16	14	9	189
**Gobitidae**													
*Chromobotia macrachantus*	0	0	0	1	2	2	8	5	6	0	0	0	24
*Chromobotia hymenophysa*	0	1	1	2	2	1	1	3	0	0	0	0	11
*Acanthopsis octoactinatus*	0	2	1	1	3	1	3	4	6	2	1	2	26
**Tetradontidae**													
*Tetraodon palembangensis*	3	4	2	4	5	12	18	21	18	15	12	5	119
**Anabantidae**													
*Anabas testudineus*	0	0	8	12	18	15	21	30	18	17	23	15	177
**Belontiidae**													
*Thrichogaster trichopterus*	0	0	0	0	1	2	3	3	2	5	3	2	21
***Channidae***													
*Channa lucius*	12	14	10	16	21	25	28	31	38	12	9	5	221
*Channa striata*	19	21	17	25	26	32	38	40	32	29	17	9	305
*Channa micropeltes*	0	0	1	2	0	4	6	7	5	1	1	0	27
*Channa pleurothalmus*	0	0	0	0	0	0	2	2	0	1	0	0	5
**Eleotridae**													
*Oxyeleotris marrmorata*	9	15	14	21	19	16	18	21	19	17	13	8	190
**Helostomatidae**													
*Helostoma temmincki*	0	0	0	5	8	11	15	23	29	32	15	11	149
**Mastacembelidae**													
*Mastacambelus unicolor*	3	5	6	7	8	8	6	7	12	11	8	8	89
***Osphronemidae***													
*Osphronemus gouramy*	0	0	0	4	3	6	7	10	8	13	15	9	75
**Pristolepididae**													
*Pristilepis grooti*	5	8	12	11	15	19	13	18	22	23	19	16	181
**Cygnolossidae**													
*Cygnolossus microlepis*	3	9	8	11	15	19	23	21	19	15	14	12	169
**Notopteridae**													
*Chitala lopis*	3	5	6	6	8	9	14	13	20	21	16	15	136
**Hemiramphidae**													
*Hemiramphus chrysopunctatus*	4	7	8	11	13	9	15	21	19	23	14	12	156
**Cichlidae**													
*Oreochromis niloticus*	57	61	69	62	59	60	76	65	67	80	69	61	786
**Grand total**	**437**	**484**	**534**	**635**	**670**	**754**	**848**	**873**	**854**	**772**	**641**	**515**	**8017**
Percentage population	5.45	6.04	6.66	7.92	8.36	9.41	10.58	10.89	10.65	9.63	8.00	6.42	
Total species	26	30	36	39	41	43	44	44	42	41	39	37	
Percentage diversity	59.09	68.18	81.82	88.64	93.18	95.45	100	100	95.45	93.18	88.64	86.36	

### Icthyodiversity in the upstream and downstream areas at Koto Panjang Reservoir

A total of 44 species belonging to 19 families and 33 genera were sampled from the four sites over one year in the upstream and downstream areas at Koto Panjang Reservoir. There were seventeen species which are commercially important, as determined by their high market value. These species including ornamental fish species such as *Chromobotia macrachantus, Chromobotia hymenophysa, Thrichogaster trichopterus* and *Mystus micracanthus*. The highest ichthyodiversity in study area was calculated during July and August 2018 (44 species), followed by June and September (42 species), May and October (41 species), April and November (39 species), December (38 species), March (36 species), February (30 species) and January (26 species). Numerically, the most abundant and diverse family was Cyprinidae, comprising of 16 species, followed by Bagridae and Channidae (represented by four species each). The fourth most diverse family was Gobitidae, represented by three species in the study area. The least diverse families were Claridae, Pangasidae, Anabantidae, Mastacembelidae, Osphronemidae, Pristolepididae, Cygnolossidae, Notopteridae, Hemiramphidae and Cichlidae, represented by only one species for each ( Table 2). *Barbodes schwanifeldi, Hemibagrus nemerus, Ompok hypophthalmus, Rasbora argyrotaenia* and *Oreochromis niloticus* were recorded as the most abundant species, comprising 5.88%, 6.20%, 6.71%, 8.76% and 9.80% of all fish caught, respectively. The least abundant were *Channa pleurothalmus*, *Hemibagrus wyckii* and *Pangasius pangasius*, representing 0.06%, 0.08% and 0.09%, respectively.

Table 3 shows different diversity indices used to calculate the species abundance data. The highest species richness was recorded between the months of June and September. Similarly, the highest values for Shannon’s diversity index (H’) were achieved during October 2018 (2.27), August and November 2018 (2.23), and the lowest values during January and February 2018 (1.74 and 1.91).

**Table 3.** Monthly diversity indices of fish fauna in the upstream and downstream areas at Koto Panjang Reservoir.

Index	Jan	Feb	Mar	Apr	May	Jun	Jul	Aug	Sep	Oct	Nov	Dec	Total
No. of individuals	437	484	534	635	670	754	848	873	854	772	641	515	8017
Richness (S)	26	30	36	39	41	43	44	44	42	41	39	37	44
Simpson (1/D)	0.25	0.22	0.22	0.23	0.22	0.22	0.20	0.19	0.19	0.18	0.18	0.20	0.21
Shannon’s (H’)	1.74	1.91	2.14	2.00	2.07	2.06	2.16	2.23	2.21	2.27	2.23	2.17	2.10
Evenness (e ˆH/S)	0.22	0.22	0.24	0.19	0.19	0.18	0.19	0.21	0.21	0.24	0.23	0.24	0.19

The highest Simpson diversity index (I/D) was recorded during the months of January (0.25), followed by April (0.23), February, March and May (0.22), and the lowest value was recorded in the months of October and November (0.18). Similarly, the highest values of species evenness (H/S) was recorded in the month of March > December and its least value was recorded during the month of June. Furthermore, the detailed values of different diversity indices on the basis of sampling sites were given in the Table 4. Whereas, the Sorenson’s coefficient (CC) between upstream and downstream areas at Koto Panjang Reservoir were presented in Table 5. The commercially important fish species captured in downstream Reservoir were *Hemibagrus wyckii*, *Hemibagrus nemurus*, *Wallago leerii*, *Pangasius pangasius*, *Osphronemus gourami*, *Puntioplites bulu*, *Rasbora argyrotaenia*, *Channa striata*, and *Channa micropeltes*. Whereas, in the upstream Reservoir found were *Pristilepis grooti*, *Oxyeleotris marrmorata*, *Hemibagrus nemurus* and *Channa striata* and *Oreocromis niloticus*.

**Table 4.** Site based diversity indices of study area in the upstream and downstream areas at Koto Panjang Reservoir.

Index	KM	BB	RB	KK	Total
No. of individuals	1701	1370	2135	2811	8017
Richness (S)	18	21	37	35	44
Simpson (1/D)	0.31	0.18	0.22	0.20	0.21
Shanon,s (H’)	1.51	1.95	2.00	2.03	2.10
Eveness_ e ˆH/S	0.25	0.33	0.20	0.22	0.19

Note: KM = Koto Mesjid, BB = Batu Bersurat, RB = Rantau Berangin, KK = Kuok.

**Table 5.** Sorenson’s coefficient (CC) between sites in the upstream and downstream areas of Koto Panjang Reservoir.

Site	KM	BB	RB	KK
KM				
BB	0.92			
RB	0.65	0.54		
KK	0.57	0.56	0.80	

Note: KM = Koto Mesjid, BB = Batu Bersurat, RB = Rantau Berangin, KK = Kuok.

**Figure 1.**
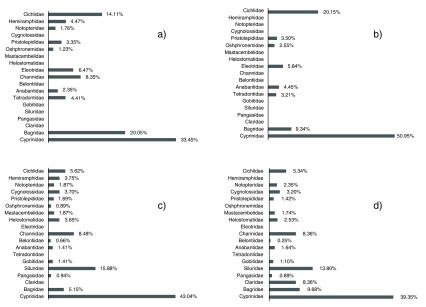
Abundance and composition of aquatic fauna in the upstream and downstream reservoir. **a**) Koto Mesjid, **b**) Batu Bersurat, **c**) Rantau Berangin and **d**) Kuok.

**Figure 2.**
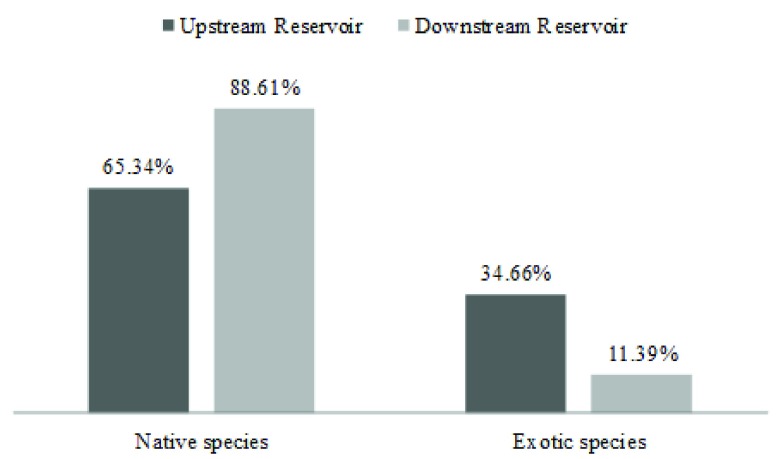
Percentage of individuals of native and exotic fish species in upstream (Koto Mesjid, KM, and Batu Bersurat, BB) and downstream reservoirs areas (Rantau Berangin, RB, and Kuok, KK).

The abundance and number of families found between sites was varied. At KM and BB (upstream reservoir), 8 and 11 families were found, respectively, while at RB and KK (downstream reservoir), 16 and 15 families were found, respectively ( Figure 1). The dominant families in each site was Cyprinidae, comprising 33.45%, 50.95%, 43.04% and 39.35% of all fish caught at KM, BB, RB and KK, respectively. Whereas, the exotic species, especially Nile tilapia ( *O. niloticus*), in each site comprised 14.11%, 20.15%, 5.62% and 5.34% of all fish caught at KM, BB, RB and KK, respectively. Some differences were also noted between the upstream and downstream reservoirs areas ( Figure 2), with a slight increase of exotic species in the upstream reservoir (from 11.39% to 34.66%) and a corresponding decrease of native species (from 88.61% to 65.34%). ****

## Discussion

Our results showed that the highest abundance and diversity of fish species collected in June, July and August, which may be due to a lesser water current in the study area during these dry season months. According to Kriaučiūnienė *et al*. ^22^ the abundance of fish species in rivers can be affected by river discharge. However, future alterations in river water temperature will have a significantly larger influence on the abundance of fish than river discharge. Overexploitation and illegal sand mining have affected the abundance of fish in the Kampar Kanan river ^23^. Furthermore, the fundamental measures of aquatic ecosystems, including species richness and diversity indices, are influenced by the alterations in abiotic factors such as river water temperature and discharge ^24– 27^.

Our study also revealed significantly lower species diversity in KM and BB (upstream reservoir) compared to that of RB and KK (downstream reservoir). The overall richness in KM was much lower than that found in RB (18 vs. 37). During the research period, we also recorded a slight increase in exotic species in the upstream reservoir and a decrease in native species. Therefore, the implication of exotic species such as Nile tilapia and common carp have negative effect to native species in the upstream area of Koto Panjang Reservoir. In contrast, in a Portuguese reservoir, it was found that there was a slight increase in exotic species in the downstream reservoir ^6^. This result might be a consequence of cumulative impacts of cultivation of fish in floating net cages, such as Nile tilapia and common carp. According to Russel *et al.* ^28^, Nile tilapia cause the extinction of native fish species by preying on eggs, fry and small fish of other species. On the other hand, the decrease in species richness can be influenced by aquaculture activity, such as water quality degradation, intensified competition, invasive species, and habitat fragmentation ^14, 29– 31^. Koto Panjang Reservoir was categorized as eutrophic, with level index values ranging from 4.6-5.2 ^32^. According to Edwards ^33^, species with high environmental tolerance would be survive in poor environmental conditions, such as high pollution caused by food waste at the reservoir, while the sensitive species will disappear. Furthermore, fish populations in a reservoir can be affected by hydropower dams ^5, 6^. In addition, the intensifying competition for food and space between wild species and the large number of cultured species will lead to a decrease in wild fish numbers ^34, 35^.

Our study confirmed the existence of three species, *Pangasius pangasius*, *Wallago leerii* and *Chitala hypselonotus*, at Koto Panjang Reservoir that were not found previously by Warsa *et al.* ^36^ and Krismono *et al.* ^37^. The study area also represents the area of the Kampar Kanan river with the largest fish species, including *W. leerii*, *H. wyckii* and *P. pangasius*. The construction of the dam for power generation purposes is posing serious threats to the biodiversity of Kampar Kanan river. After the construction of the Koto Panjang dam on Kampar Kanan river, the movement of fish upstream has been restricted. *Osteochilus kelabau* has not been reported at RB in our study although this species has previously been found in this area ^4^. Similarly, the population of *H. wyckii* has also been dramatically reduced and restricted between Koto Panjang Reservoir barrages and Kampar Kanan river after the construction of these barrages. *H. wyckii* in the Kampar Kanan river is categorized as ‘vulnerable to endangered’ ^38^. According to Piria *et al.* ^39^, the disturbances to the fish assemblage pattern have coincided with the presence of multiple stressors of human origin, such as pollution, flood protection and dam construction. Meanwhile, dams can of change the hydrological dynamics, patterns of biological production, loss of native species in the downstream regions and distribution of organisms in space and time ^5, 7, 12, 40, 41^.

There are a number of inadvertently introduced fish species in the upstream and downstream Reservoir, such as *O. niloticus*, *Cyprinus carpio*, *Leptobarbus hoevenii* and *Pangasianodan hypophthalmus,* while rest of the 40 species belong to the native fish fauna of the Kampar Kanan river ^4^. The unique feature of the abundance data was that the exotic family Cichlidae is well established and its population is increasing day by day, especially in the upstream areas at Koto Panjang Reservoir. In recent years, the Koto Panjang Reservoir has had very important roles, such as housing power plants with a capacity of 114 MW and serving as a fishery capture and aquaculture area with floating net cage farming, especially for the cultivation of *O. niloticus.* Cichlidae was the third most abundant family. These alien fish species represent a significant risk for the local fish community and other aquatic animals. Gu *et al.* ^31^ state that the invasion of *O. niloticus* negatively affected the fishery economy and native fish species in the Pearl River of Guangdong Province, China.

In addition to the species richness (S) analysis, Shannon diversity index (H’), Simpson diversity index (D) and evenness (H/S), we also analyzed the Sorenson’s coefficient (CC) between the upstream and downstream sites. The Sorenson’s coefficient for each site varied between 0.54 and 0.92. The highest value of Sorenson’s coefficient was recorded between KM and RB, at 0.92, and the lowest value was recorded between BB and RB, at 0.54 ( Table 5). According to Sorenson’s coefficient, these communities have quite a bit of overlap or similarity.

The high diversity of native species in the water body will decrease their tolerance in poor aquatic environments. In contrast, the invasive fish species have a high tolerance to poor water quality ^42^. The interaction between different species, combined with the limnological and physical properties of the aquatic ecosystem, may influence the diversity and distribution of fish fauna ^43^. In this study, the diversity of fish to be found smaller than Fhitra and Siregar ^3^, who reported 58 fish species belonging to 23 families and 40 genera from Kampar Kanan river. Simanjuntak *et al.* ^44^ described 86 species and 21 families in the Kampar Kiri river of the Kampar District. On the other hand, Nurdawati *et al.* ^45^ reported 96 species in the Batanghari river, Indonesia. Additionally, Bahri ^46^ described 86 species in the Musi river and Kottelat and Whitten ^47^ reported 1300 species of freshwater fish across Indonesia that live in wetlands (rivers, lakes, bogs, oxbow lakes, floodplains, etc.).

## Conclusion

The results indicate that the diversity and distribution of fish fauna in upstream and downstream areas of the Koto Panjang Reservoir were varied, and the evenness was low. The abundance and composition of fish in each site was dominated by Cyprinidae families, although exotic species were more dominant in the upstream reservoir compared to the downstream reservoir areas. Therefore, the management of the river and reservoir in a more holistic manner is important, for example, the management of land use, sand mining and aquaculture activity, as well as possible habitat restorations. All the factors above are a prerequisite for the environmental sustainability and conservation of fish diversity in the upstream and downstream areas at Koto Panjang reservoir and other regions.

## Data availability

### Underlying data

Figshare: Row data fish fauna at upstream and downstream. https://doi.org/10.6084/m9.figshare.8964284.v1 ^20^

This project contains the following underlying data:

– Tables 2 – 5 (raw data for ichthyodiversity of fish in each site).
– Table 6 (data for abundance and composition of aquatic fauna each sites in the upstream and downstream areas at Koto Panjang Reservoir)
– Table 7 (data for grand total and percentage of families of aquatic fauna in the upstream areas at Koto Panjang Reservoir)
– Table 8 (data for grand total and percentage of families of aquatic fauna in the downstream areas at Koto Panjang Reservoir)
– Table 9 (character morphometric and meristic of fishes of upstream and downstream areas at Koto Panjang Reservoir, Riau Province-Indonesia)
– Table 10 (sample sizes of fish populations (n=10) in the upstream and downstream areas at Koto Panjang Reservoir on January to December 2018)

Data are available under the terms of the Creative Commons Attribution 4.0 International license (CC-BY 4.0).
