# Supplementary material for: Diversity and distribution of fish fauna of upstream and downstream areas at Koto Panjang Reservoir, Riau Province, Indonesia
Source: F1000Res. 2020 Feb 5;8:1435. Originally published 2019 Aug 14. [Version 2] doi: 10.12688/f1000research.19679.2 (PMC7029753; doi:10.12688/f1000research.19679.2)
Supplement: Peer review history

# Peer review: Reviewer response for version 2

**DOI:** 10.5256/f1000research.23739.r59572

**Published:** 2020-02-17

**Authors:** Rudy Agung Nugroho

**Affiliations:** 1 Animal Physiology, Development and Molecular Laboratory, Department of Biology, Faculty of Mathematic and Natural Science, Mulawarman University, Samarinda, Indonesia

Based on the revised manuscript, I can judge that the article is now better than previous version.

 This article can be indexed to improve the visibility and citations.

Is the work clearly and accurately presented and does it cite the current literature?

Yes

If applicable, is the statistical analysis and its interpretation appropriate?

Yes

Are all the source data underlying the results available to ensure full reproducibility?

Yes

Is the study design appropriate and is the work technically sound?

Partly

Are the conclusions drawn adequately supported by the results?

Yes

Are sufficient details of methods and analysis provided to allow replication by others?

Partly

Reviewer Expertise:

My area research is about fish biology, animal physiology, fish nutrition, and fish conservation.

I confirm that I have read this submission and believe that I have an appropriate level of expertise to confirm that it is of an acceptable scientific standard.

# Peer review: Reviewer response for version 2

**DOI:** 10.5256/f1000research.23739.r59573

**Published:** 2020-02-11

**Authors:** Christopher Marlowe A. Caipang

**Affiliations:** 1 College of Liberal Arts, Sciences and Education, University of San Agustin, Iloilo City, Philippines

I have read the revised version of the manuscript and the authors have addressed the issues that I raised in the ms. I think that this is acceptable for indexing.

Is the work clearly and accurately presented and does it cite the current literature?

Partly

If applicable, is the statistical analysis and its interpretation appropriate?

Not applicable

Are all the source data underlying the results available to ensure full reproducibility?

Yes

Is the study design appropriate and is the work technically sound?

Partly

Are the conclusions drawn adequately supported by the results?

Yes

Are sufficient details of methods and analysis provided to allow replication by others?

Yes

Reviewer Expertise:

NA

I confirm that I have read this submission and believe that I have an appropriate level of expertise to confirm that it is of an acceptable scientific standard.

# Peer review: Reviewer response for version 1

**DOI:** 10.5256/f1000research.21583.r56362

**Published:** 2019-11-25

**Authors:** Christopher Marlowe A. Caipang

**Affiliations:** 1 College of Liberal Arts, Sciences and Education, University of San Agustin, Iloilo City, Philippines

The manuscript presented the results in a straightforward manner. The Introduction should contain studies that had been conducted in the area and the research gap that the authors need to address.

 Perhaps the authors could have done statistical analyses to compare the diversity in the sites where the data have been obtained.

 In the Discussion section, the authors must clearly explain the implications of their study and the impacts of the results that they had in terms of managing the resources.

 I confirm that I have read this submission and believe that I have an appropriate level of expertise to confirm that it is of an acceptable scientific standard, however I have significant reservations, as outlined above.

Is the work clearly and accurately presented and does it cite the current literature?

Partly

If applicable, is the statistical analysis and its interpretation appropriate?

Not applicable

Are all the source data underlying the results available to ensure full reproducibility?

Yes

Is the study design appropriate and is the work technically sound?

Partly

Are the conclusions drawn adequately supported by the results?

Yes

Are sufficient details of methods and analysis provided to allow replication by others?

Yes

Reviewer Expertise:

Aquaculture; Aquatic biotechnology; health management

I confirm that I have read this submission and believe that I have an appropriate level of expertise to confirm that it is of an acceptable scientific standard, however I have significant reservations, as outlined above.

# Response

**Published:** 2019-11-29

**Authors:** Netti Aryani

**Affiliations:** Universitas Riau, Indonesia

**Response for Christopher Marlowe A. Caipang Comments:**

**Introduction: **We have been added new references in introduction who conducted research in this area.

**Methods: **We do not use statistical analysis for calculate the data. All the data was entered into excel 2010 (version 14.0). Then, the data of different species for abundance and occurrence was calculated based on the equation of Shannon diversity Index, Simpson diversity index and Sorenson’s coefficient (CC)

**Discussion**: we have been added the implication of our study and effect to managing fish resources. Such as the implication of exotic species for examples Nile tilapia and common carp have negative effect to native species in the upstream area of Koto Panjang Reservoir.

# Peer review: Reviewer response for version 1

**DOI:** 10.5256/f1000research.21583.r52476

**Published:** 2019-09-02

**Authors:** Rudy Agung Nugroho

**Affiliations:** 1 Animal Physiology, Development and Molecular Laboratory, Department of Biology, Faculty of Mathematic and Natural Science, Mulawarman University, Samarinda, Indonesia

In general this project is useful to add important information regarding fish fauna in Indonesia. However, some part in the article, need to be clarified by the author(s). Please see comments below:

**Abstract**

 Conclusion: *Nile tilapia* should be mentioned as exotic species which found in upstream, cause it is a dominant species.

**Introduction**

1. "In 2015, the total ﬁshery production of Indonesia was 16,954,344 metric tons, of which 455,270 metrics tons were obtained from inland capture ﬁsheries, 6,065,060 metric tons were obtained from marine fisheries, and 10,074,014 metric tons were obtained from aquaculture fisheries production". Use newest statistic data would be better, instead of 2015.

**Methods**

1. Though endangered species  were returned to the river. Please explain on how the author caught this fish? Doesn’t hurt the fish?
2. Please also explain on how fish euthanization? Does all fish same treatment using piercing part on their brain?
3. It is stated in the methods that "the length, body weight and morphometric characteristics were only collected for 10 individual specimens from each species. This mean in total 10 individual specimens from over a year? Please explain, because there are some species that have number less than 10 each month, for example *Hemibagrus wyckii.*

**Results**

1. The name *Pangasius hypophthalmus *in the Table 2, should be changed to *Pangasianodon hypophthalmus* *.* Please check fishbase.org here. 
2. Please also check the fish name for *Pangasius pangasius.*

Overall, this article is well written and has good structure.

Is the work clearly and accurately presented and does it cite the current literature?

Yes

If applicable, is the statistical analysis and its interpretation appropriate?

Yes

Are all the source data underlying the results available to ensure full reproducibility?

Yes

Is the study design appropriate and is the work technically sound?

Partly

Are the conclusions drawn adequately supported by the results?

Yes

Are sufficient details of methods and analysis provided to allow replication by others?

Partly

Reviewer Expertise:

My area research is about fish biology, animal physiology, fish nutrition, and fish conservation.

I confirm that I have read this submission and believe that I have an appropriate level of expertise to confirm that it is of an acceptable scientific standard, however I have significant reservations, as outlined above.

# Response

**Published:** 2019-09-09

**Authors:** Netti Aryani

**Affiliations:** Universitas Riau, Indonesia

**Response for Rudy Agung Nugroho Comments:**

**Abstract** : We would like to clarify that “Nile tilapia as exotic species have been mention in the point of results.

**Introduction** : the latest data we have in the year 2015, there is no recent data have been found from Indonesian Fisheries Statistics.

**Methods **:

1. We caught the fish using fishing pole. We have explained in the methods
2. We have been explained in the Methods, yes…all fish are same treatment except for endangered species.
3. Fish caught more than 10 individuals for a year, morphometric measurement for only 10 individual of fish. However, fish caught less than 10 individual, the morphometric analysis were measured for all fish.

**Results** :

1. We have been check in the fish base. The name of *Pangasius pangasius* is correct.

2. The name of *Pangasius pangasius* is correct. Fishbase.org said that *Pangasius pangasius* spesies for aquaculture in Indonesia, Thailand, and Malaysia. However, *Pangasianodon hypophthalmus* species was categorize as endangered species and found in Mekong, Chao Phraya, and Maeklong basins.
